# Stigma and increase of leprosy cases in SouthEast Sulawesi Province, Indonesia

**DOI:** 10.4314/ahs.v18i1.5

**Published:** 2018-03

**Authors:** Ramadhan Tosepu, Joko Gunawan, Devi Savitri Effendy, Fitri Rachmillah Fadmi

**Affiliations:** 1 Faculty of Public Health, University of Halu Oleo Kendari, Indonesia; 2 Akademi Keperawatan Pemerintah Kabupaten Belitung, Indonesia; 3 Sekolah Tinggi Ilmu Kesehatan Mandala Waluya Kendari, Indonesia

Dear Editor,

Leprosy remains an important health issue. The disease is characterised by a chronic granulomatous infection of the skin and peripheral nerves caused by *Mycobacterium leprae*. The disease is classified into two, namely paucibacillary (PB) or multibacillary (MB) leprosy. PB leprosy is a milder form of disease, characterized by several (ie, up to five) hypopigmented, pale and reddish lesions, and hypo- or anesthetic lesions. MB leprosy is associated with several skin lesions that manifest as nodules, plaques, or diffuse skin infiltration^1^–^3^.

Globally, India, Brazil, and Indonesia are the three countries with scattered populations reporting over 10,000 (81%) of new patients each year, which indicated that the world is demanded to make a great progress towards the goal of eliminating leprosy^4^. In this short article, the authors report the leprocy cases in SouthEast Sulawesi province as the highest case of leprosy in Indonesia.

**Figure:**
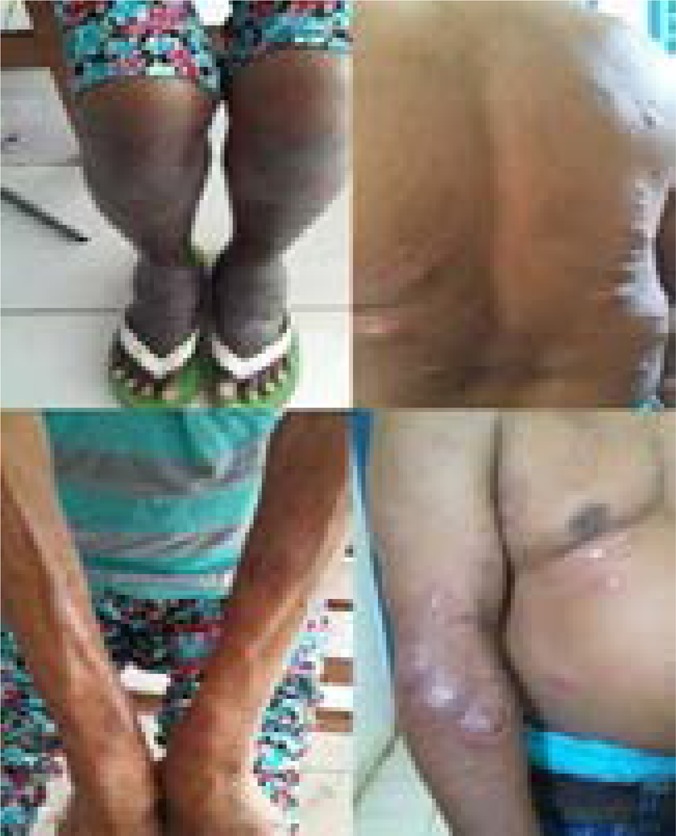


**Figure 1:**
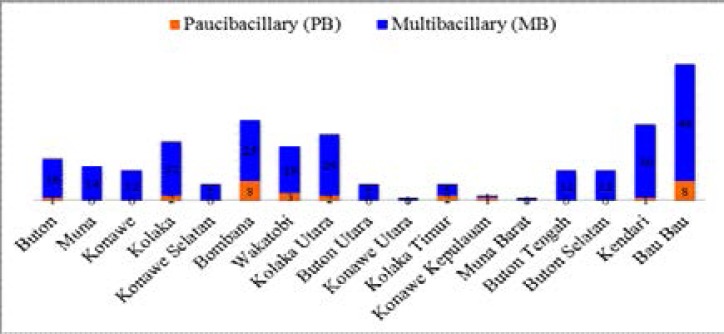
The new cases based on the leprosy type Southeast Sulawesi province (2015) (5)

**Figure 2:**
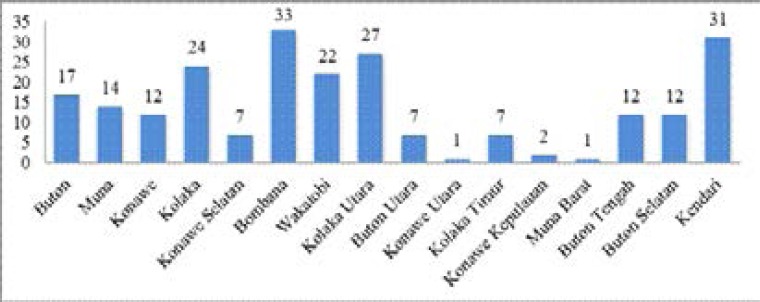
Distribution of leprosy by District in Southeast Sulawesi Province, Indonesia in 2015 (5)

In 2015, the prevalence of leprosy in the province was1.25 / 10.000 population, with new 275 cases of MB leprosy and 28 new cases of PB leprocy (see Figure 1)^5^. The spread of the disease is present in all districts in the province of SouthEast Sulawesi (see Figure 2). This condition however resulted in great stress on the community due to the stigma in the province.

Although leprosy is the oldest disease known to man^6^, leprosy-related stigma is still very strong in the society in the SouthEast sulawesi Indonesia. It is believed to be inherited and associated with ideas of unclean blood, which is shameful and not accepted normally in society. However, that stigma affects the lives of the people affected by leprosy and inhibits the treatment process, and might lead to progressive leprosy, resulting in permanent damage to the skin, nerves, limbs and eyes^3^. Some studies have concluded that stigma affects many aspects of the lives of people affected by leprosy including mobility, interpersonal relationships, marriage, employment, leisure activities, and attendance at social and religious functions^7^. If the stigma about leprosy is not changed, then it will be difficult to eliminate leprosy as a public health problem and the incidence might be increased.

Thus, this condition calls for the role of public health to set up the strategy in Indonesia, particularly in the province of SouthEast sulawesi to eliminate the increase of leprosy and to fight stigma. These focused on 1) Increasing early case detection in the community; 2) Improving the quality of leprosy services, including rehabilitation services that are integrated with basic health care and referrals; 3) Changing the image of the disease that leprosy is curable by disseminating information or media campaigns; 4) The elimination of stigma by increasing the knowledge of the disease; 5) Empowering those who have experienced leprosy in various aspects of life, and strengthening their participation in controlling leprosy; 6) Restoring self-esteem; and 7) Developing program that prevents disabilities or permanent damage^8^.
